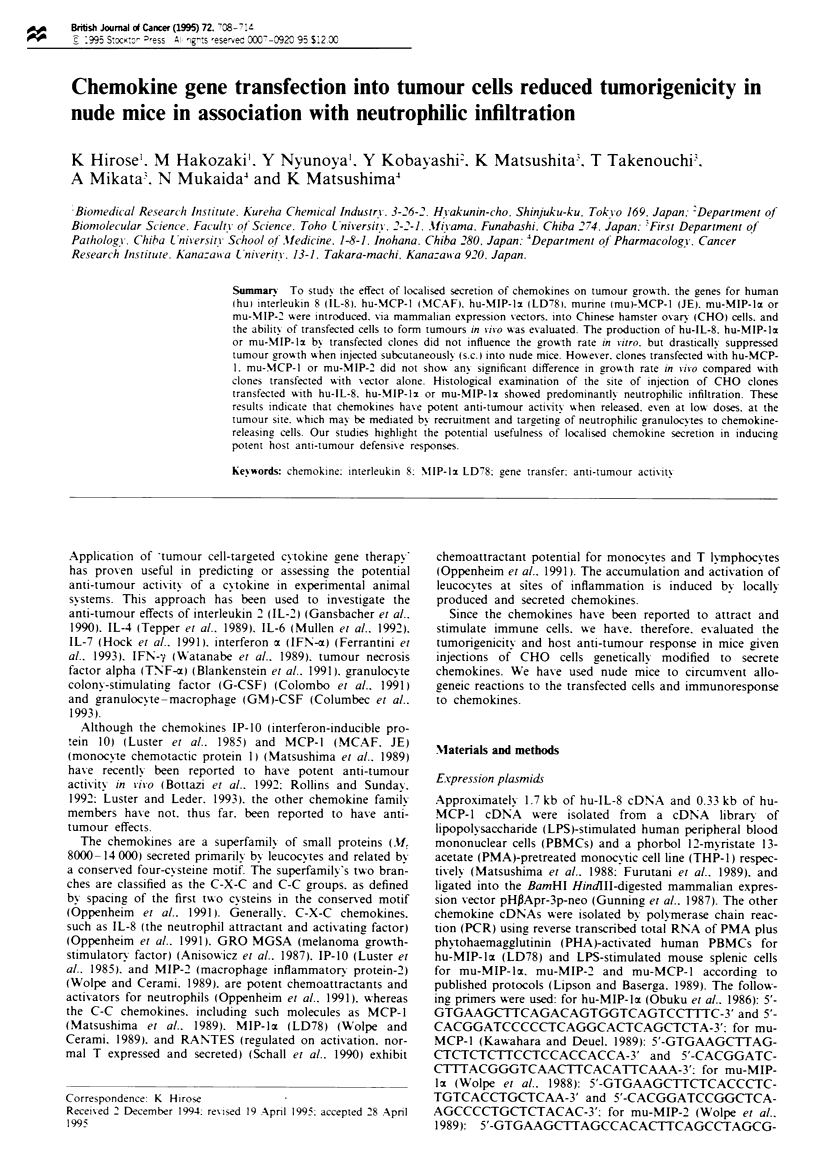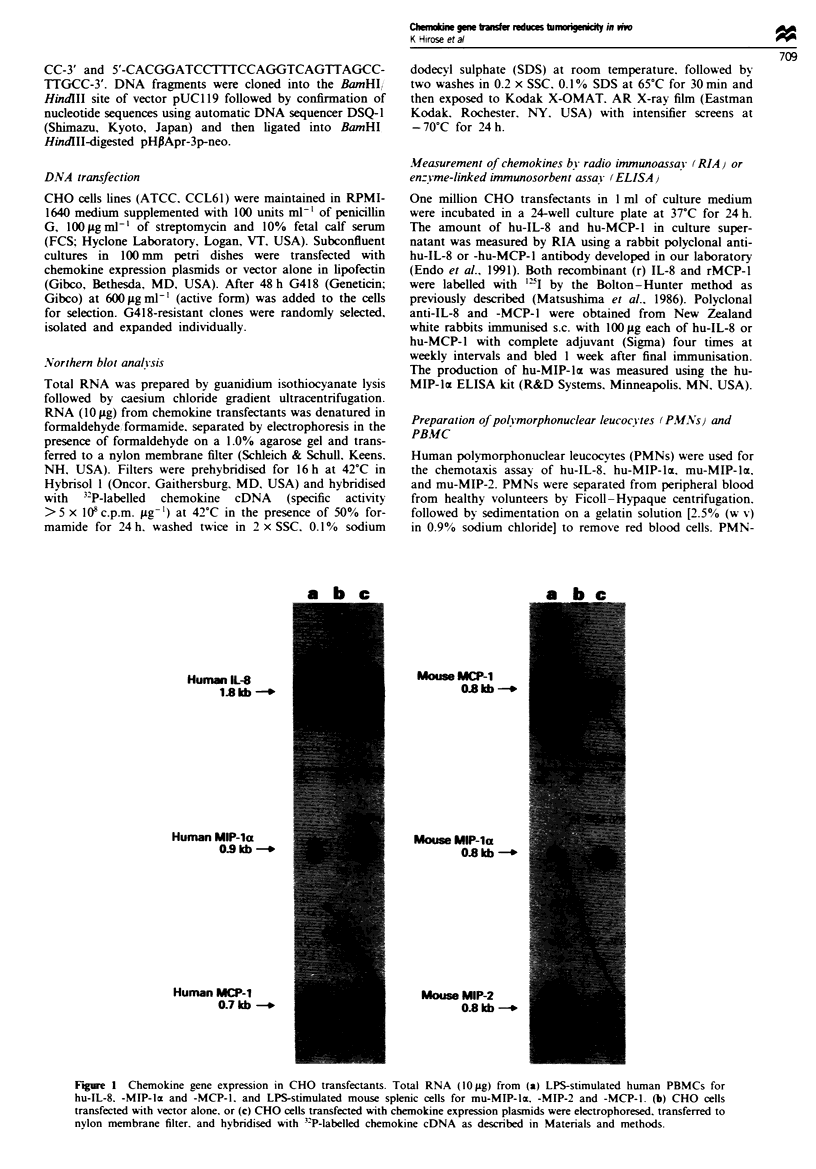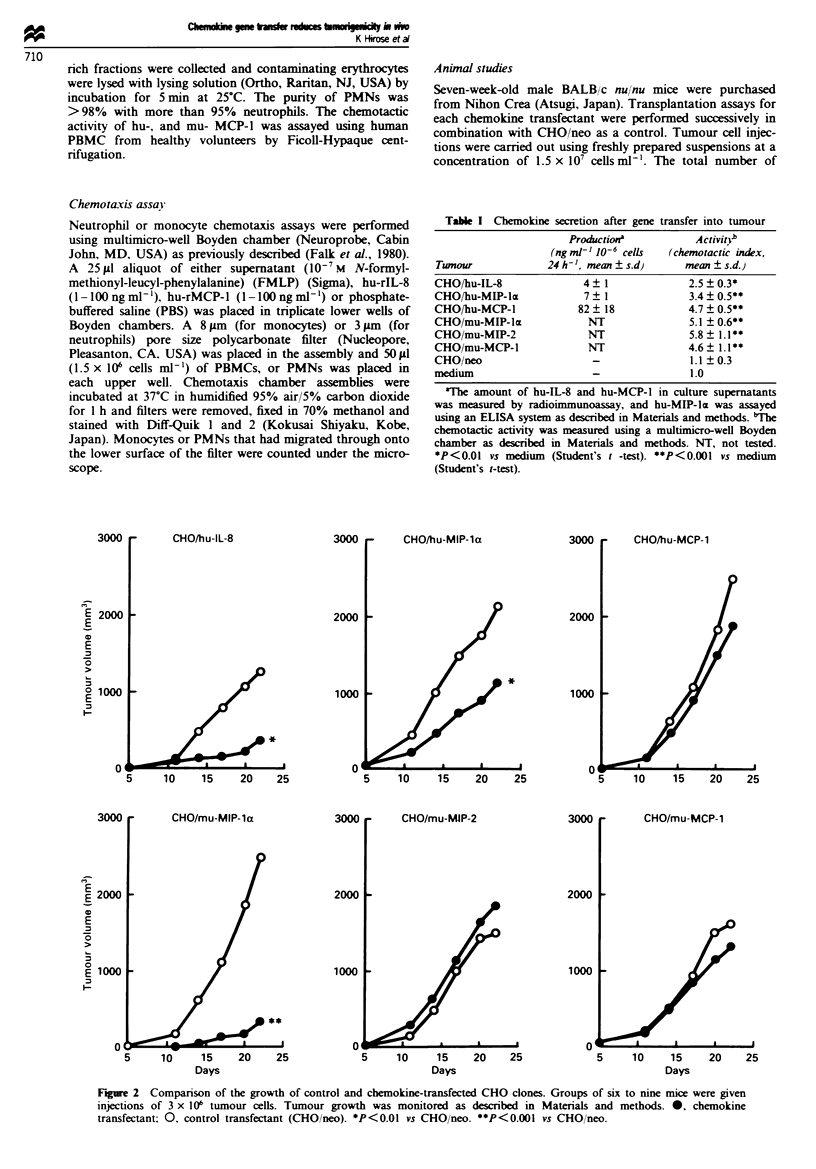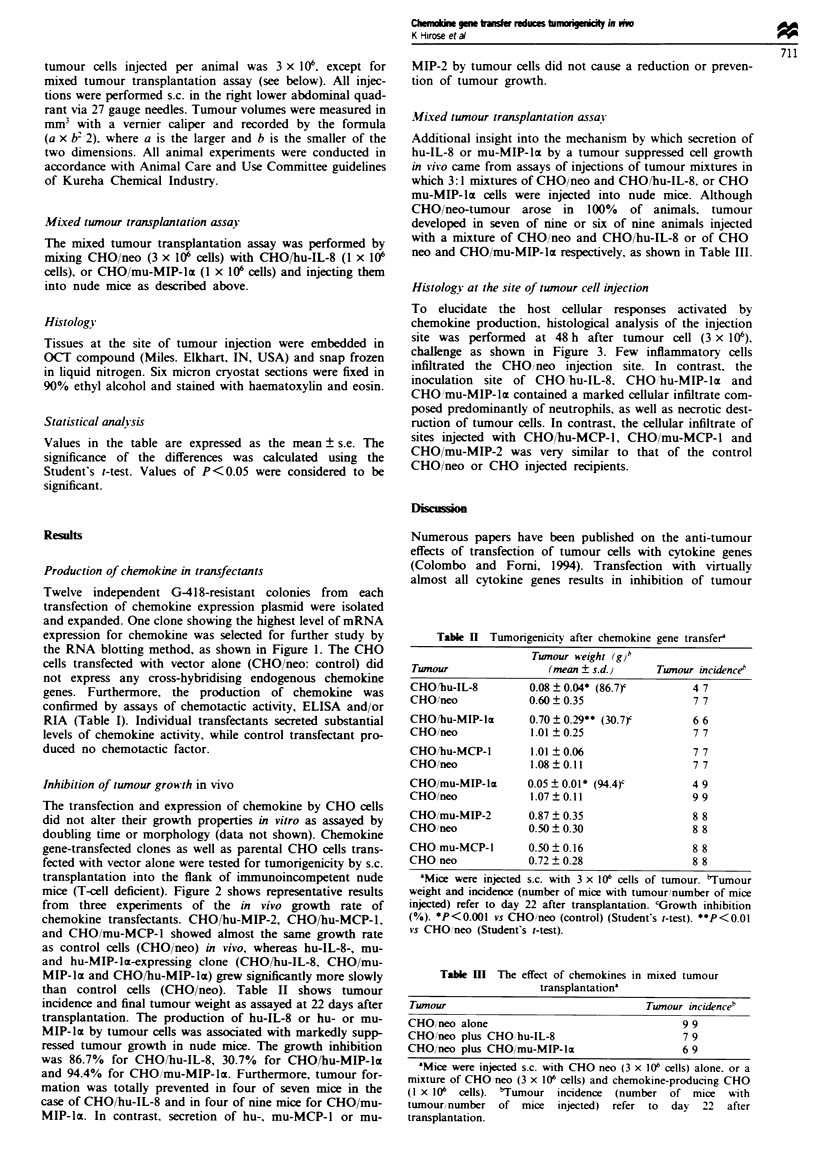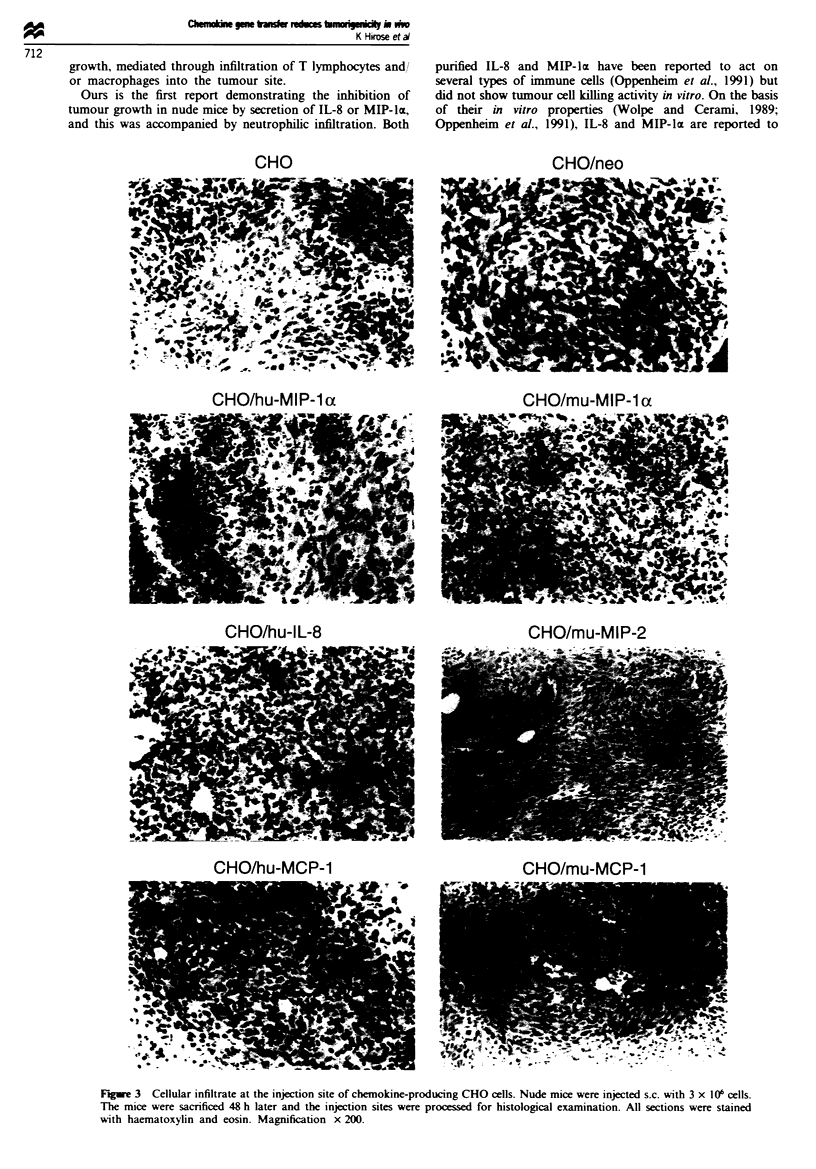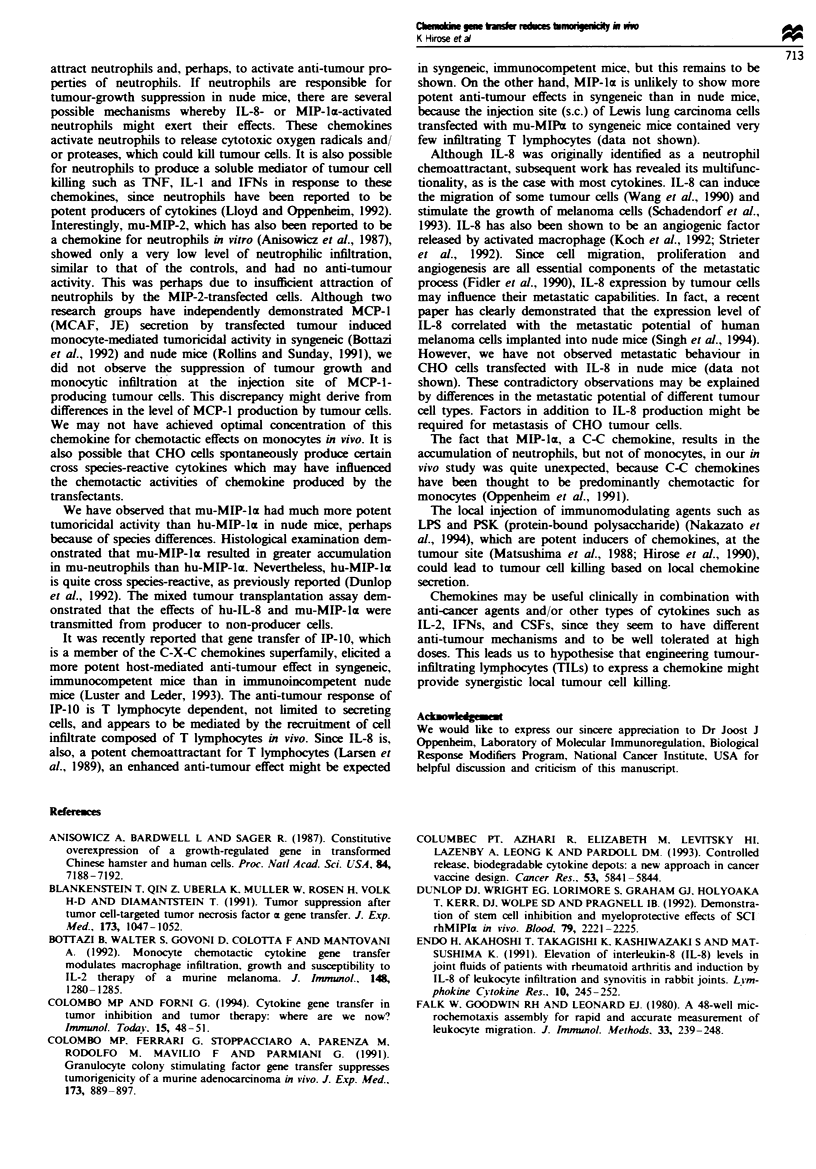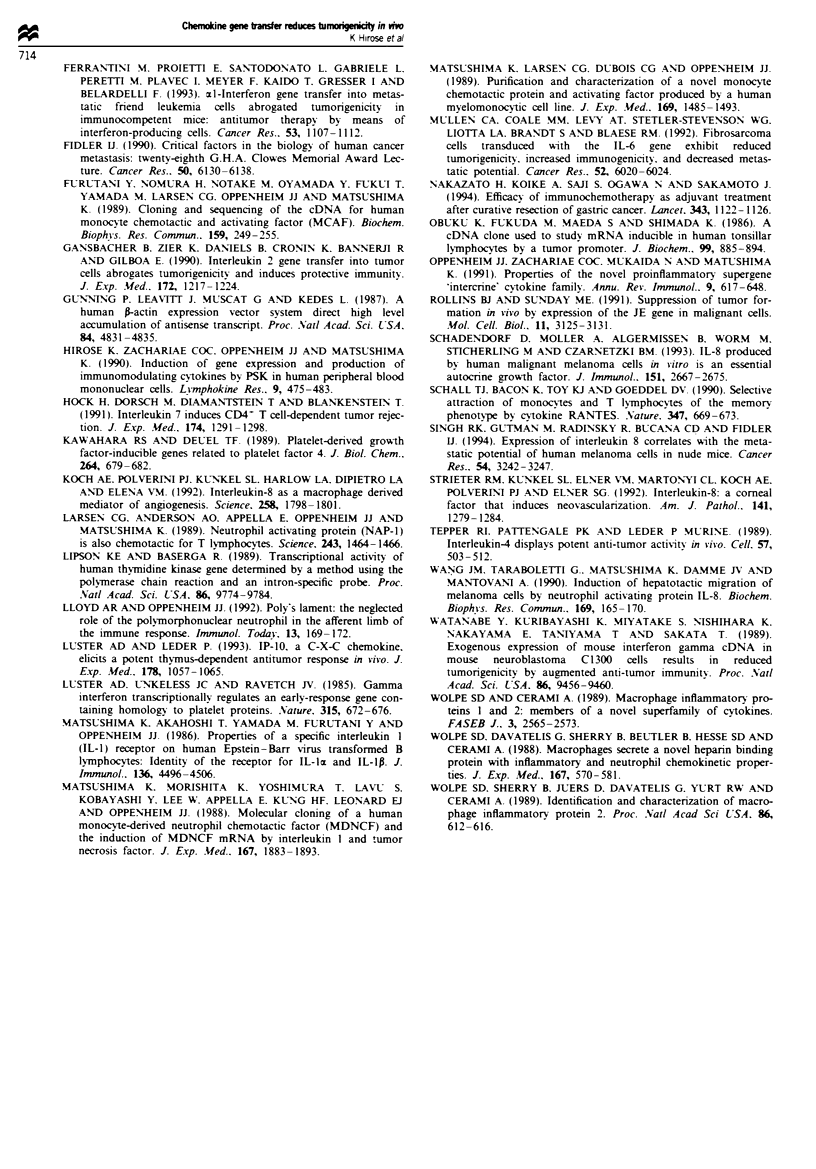# Chemokine gene transfection into tumour cells reduced tumorigenicity in nude mice in association with neutrophilic infiltration.

**DOI:** 10.1038/bjc.1995.398

**Published:** 1995-09

**Authors:** K. Hirose, M. Hakozaki, Y. Nyunoya, Y. Kobayashi, K. Matsushita, T. Takenouchi, A. Mikata, N. Mukaida, K. Matsushima

**Affiliations:** Biomedical Research Institute, Kureha Chemical Industry, Tokyo, Japan.

## Abstract

**Images:**


British Journal of Cancer (1995) 72, 708-714

© 1995 Stockton Press All rights reserved 0007-0920/95 $12.00

### Chemokine gene transfection into tumour cells reduced tumorigenicity in nude mice in association with neutrophilic infiltration

K Hirose[1], M Hakozaki[1], Y Nyunoya[1], Y Kobayashi[2], K Matsushita[3], T Takenouchi[3], A Mikata[3], N Mukaida[4] and K Matsushima[4]

[1]Biomedical Research Institute, Kureha Chemical Industry, 3-26-2, Hyakunin-cho, Shinjuku-ku, Tokyo 169, Japan; [2]Department of Biomolecular Science, Faculty of Science, Toho University, 2-2-1, Miyama, Funabashi, Chiba 274, Japan; [3]First Department of Pathology, Chiba University School of Medicine, 1-8-1, Inohana, Chiba 280, Japan; [4]Department of Pharmacology, Cancer Research Institute, Kanazawa Univerity, 13-1, Takara-machi, Kanazawa 920, Japan.

**Summary** To study the effect of localised secretion of chemokines on tumour growth, the genes for human (hu) interleukin 8 (IL-8), hu-MCP-1 (MCAF), hu-MIP-1α (LD78), murine (mu)-MCP-1 (JE), mu-MIP-1α or mu-MIP-2 were introduced, via mammalian expression vectors, into Chinese hamster ovary (CHO) cells, and the ability of transfected cells to form tumours *in vivo* was evaluated. The production of hu-IL-8, hu-MIP-1α or mu-MIP-1α by transfected clones did not influence the growth rate *in vitro*, but drastically suppressed tumour growth when injected subcutaneously (s.c.) into nude mice. However, clones transfected with hu-MCP-1, mu-MCP-1 or mu-MIP-2 did not show any significant difference in growth rate *in vivo* compared with clones transfected with vector alone. Histological examination of the site of injection of CHO clones transfected with hu-IL-8, hu-MIP-1α or mu-MIP-1α showed predominantly neutrophilic infiltration. These results indicate that chemokines have potent anti-tumour activity when released, even at low doses, at the tumour site, which may be mediated by recruitment and targeting of neutrophilic granulocytes to chemokine-releasing cells. Our studies highlight the potential usefulness of localised chemokine secretion in inducing potent host anti-tumour defensive responses.

**Keywords:** chemokine; interleukin 8; MIP-1α LD78; gene transfer; anti-tumour activity

Application of 'tumour cell-targeted cytokine gene therapy' has proven useful in predicting or assessing the potential anti-tumour activity of a cytokine in experimental animal systems. This approach has been used to investigate the anti-tumour effects of interleukin 2 (IL-2) (Gansbacher *et al.*, 1990), IL-4 (Tepper *et al.*, 1989), IL-6 (Mullen *et al.*, 1992), IL-7 (Hock *et al.*, 1991), interferon α (IFN-α) (Ferrantini *et al.*, 1993), IFN-γ (Watanabe *et al.*, 1989), tumour necrosis factor alpha (TNF-α) (Blankenstein *et al.*, 1991), granulocyte colony-stimulating factor (G-CSF) (Colombo *et al.*, 1991) and granulocyte-macrophage (GM)-CSF (Columbec *et al.*, 1993).

Although the chemokines IP-10 (interferon-inducible protein 10) (Luster *et al.*, 1985) and MCP-1 (MCAF, JE) (monocyte chemotactic protein 1) (Matsushima *et al.*, 1989) have recently been reported to have potent anti-tumour activity *in vivo* (Bottazi *et al.*, 1992; Rollins and Sunday, 1992; Luster and Leder, 1993), the other chemokine family members have not, thus far, been reported to have anti-tumour effects.

The chemokines are a superfamily of small proteins ($M_r$ 8000-14 000) secreted primarily by leucocytes and related by a conserved four-cysteine motif. The superfamily's two branches are classified as the C-X-C and C-C groups, as defined by spacing of the first two cysteins in the conserved motif (Oppenheim *et al.*, 1991). Generally, C-X-C chemokines, such as IL-8 (the neutrophil attractant and activating factor) (Oppenheim *et al.*, 1991), GRO MGSA (melanoma growth-stimulatory factor) (Anisowicz *et al.*, 1987), IP-10 (Luster *et al.*, 1985), and MIP-2 (macrophage inflammatory protein-2) (Wolpe and Cerami, 1989), are potent chemoattractants and activators for neutrophils (Oppenheim *et al.*, 1991), whereas the C-C chemokines, including such molecules as MCP-1 (Matsushima *et al.*, 1989), MIP-1α (LD78) (Wolpe and Cerami, 1989), and RANTES (regulated on activation, normal T expressed and secreted) (Schall *et al.*, 1990) exhibit chemoattractant potential for monocytes and T lymphocytes (Oppenheim *et al.*, 1991). The accumulation and activation of leucocytes at sites of inflammation is induced by locally produced and secreted chemokines.

Since the chemokines have been reported to attract and stimulate immune cells, we have, therefore, evaluated the tumorigenicity and host anti-tumour response in mice given injections of CHO cells genetically modified to secrete chemokines. We have used nude mice to circumvent allogeneic reactions to the transfected cells and immunoresponse to chemokines.

### Materials and methods

### *Expression plasmids*

Approximately 1.7 kb of hu-IL-8 cDNA and 0.33 kb of hu-MCP-1 cDNA were isolated from a cDNA library of lipopolysaccharide (LPS)-stimulated human peripheral blood mononuclear cells (PBMCs) and a phorbol 12-myristate 13-acetate (PMA)-pretreated monocytic cell line (THP-1) respectively (Matsushima *et al.*, 1988; Furutani *et al.*, 1989), and ligated into the *Bam*HI *Hind*III-digested mammalian expression vector pHβApr-3p-neo (Gunning *et al.*, 1987). The other chemokine cDNAs were isolated by polymerase chain reaction (PCR) using reverse transcribed total RNA of PMA plus phytohaemagglutinin (PHA)-activated human PBMCs for hu-MIP-1α (LD78) and LPS-stimulated mouse splenic cells for mu-MIP-1α, mu-MIP-2 and mu-MCP-1 according to published protocols (Lipson and Baserga, 1989). The following primers were used: for hu-MIP-1α (Obuku *et al.*, 1986): 5'-GTGAAGCTTCAGACAGTGGTCAGTCCTTTC-3' and 5'-CACGGATCCCCCTCAGGCACTCAGCTCTA-3'; for mu-MCP-1 (Kawahara and Deuel, 1989): 5'-GTGAAGCTTAG-CTCTCTCTTCCTCCACCACCA-3' and 5'-CACGGATC-CTTTACGGGTCAACTTCACATTCAAA-3'; for mu-MIP-1α (Wolpe *et al.*, 1988): 5'-GTGAAGCTTCTCACCCTC-TGTCACCTGCTCAA-3' and 5'-CACGGATCCGGCTCA-AGCCCCTGCTCTACAC-3'; for mu-MIP-2 (Wolpe *et al.*, 1989): 5'-GTGAAGCTTAGCCACACTTCAGCCTAGCG-

Correspondence: K Hirose

Received 2 December 1994; revised 19 April 1995; accepted 28 April 1995

CC-3′ and 5′-CACGGATCCTTTCCAGGTCAGTTAGCC-TTGCC-3′. DNA fragments were cloned into the *Bam*HI/*Hin*dIII site of vector pUC119 followed by confirmation of nucleotide sequences using automatic DNA sequencer DSQ-1 (Shimazu, Kyoto, Japan) and then ligated into *Bam*HI/*Hin*dIII-digested pHβApr-3p-neo.

### *DNA transfection*

CHO cells lines (ATCC, CCL61) were maintained in RPMI-1640 medium supplemented with 100 units $ml^{-1}$ of penicillin G, 100 μg $ml^{-1}$ of streptomycin and 10% fetal calf serum (FCS; Hyclone Laboratory, Logan, VT, USA). Subconfluent cultures in 100 mm petri dishes were transfected with chemokine expression plasmids or vector alone in lipofectin (Gibco, Bethesda, MD, USA). After 48 h G418 (Geneticin; Gibco) at 600 μg $ml^{-1}$ (active form) was added to the cells for selection. G418-resistant clones were randomly selected, isolated and expanded individually.

### *Northern blot analysis*

Total RNA was prepared by guanidium isothiocyanate lysis followed by caesium chloride gradient ultracentrifugation. RNA (10 μg) from chemokine transfectants was denatured in formaldehyde/formamide, separated by electrophoresis in the presence of formaldehyde on a 1.0% agarose gel and transferred to a nylon membrane filter (Schleich & Schull, Keens, NH, USA). Filters were prehybridised for 16 h at 42°C in Hybrisol 1 (Oncor, Gaithersburg, MD, USA) and hybridised with $^{32}$P-labelled chemokine cDNA (specific activity $>5 \times 10^8$ c.p.m. $\mu g^{-1}$) at 42°C in the presence of 50% formamide for 24 h, washed twice in 2 × SSC, 0.1% sodium dodecyl sulphate (SDS) at room temperature, followed by two washes in 0.2 × SSC, 0.1% SDS at 65°C for 30 min and then exposed to Kodak X-OMAT, AR X-ray film (Eastman Kodak, Rochester, NY, USA) with intensifier screens at −70°C for 24 h.

### *Measurement of chemokines by radio immunoassay (RIA) or enzyme-linked immunosorbent assay (ELISA)*

One million CHO transfectants in 1 ml of culture medium were incubated in a 24-well culture plate at 37°C for 24 h. The amount of hu-IL-8 and hu-MCP-1 in culture supernatant was measured by RIA using a rabbit polyclonal anti-hu-IL-8 or -hu-MCP-1 antibody developed in our laboratory (Endo *et al*., 1991). Both recombinant (r) IL-8 and rMCP-1 were labelled with $^{125}$I by the Bolton–Hunter method as previously described (Matsushima *et al*., 1986). Polyclonal anti-IL-8 and -MCP-1 were obtained from New Zealand white rabbits immunised s.c. with 100 μg each of hu-IL-8 or hu-MCP-1 with complete adjuvant (Sigma) four times at weekly intervals and bled 1 week after final immunisation. The production of hu-MIP-1α was measured using the hu-MIP-1α ELISA kit (R&D Systems, Minneapolis, MN, USA).

### *Preparation of polymorphonuclear leucocytes (PMNs) and PBMC*

Human polymorphonuclear leucocytes (PMNs) were used for the chemotaxis assay of hu-IL-8, hu-MIP-1α, mu-MIP-1α, and mu-MIP-2. PMNs were separated from peripheral blood from healthy volunteers by Ficoll–Hypaque centrifugation, followed by sedimentation on a gelatin solution [2.5% (w/v) in 0.9% sodium chloride] to remove red blood cells. PMN-

**Figure 1** Chemokine gene expression in CHO transfectants. Total RNA (10 μg) from (**a**) LPS-stimulated human PBMCs for hu-IL-8, -MIP-1α and -MCP-1, and LPS-stimulated mouse splenic cells for mu-MIP-1α, -MIP-2 and -MCP-1. (**b**) CHO cells transfected with vector alone, or (**c**) CHO cells transfected with chemokine expression plasmids were electrophoresed, transferred to nylon membrane filter, and hybridised with $^{32}$P-labelled chemokine cDNA as described in Materials and methods.

rich fractions were collected and contaminating erythrocytes were lysed with lysing solution (Ortho, Raritan, NJ, USA) by incubation for 5 min at 25°C. The purity of PMNs was >98% with more than 95% neutrophils. The chemotactic activity of hu-, and mu- MCP-1 was assayed using human PBMC from healthy volunteers by Ficoll-Hypaque centrifugation.

*Chemotaxis assay*

Neutrophil or monocyte chemotaxis assays were performed using multimicro-well Boyden chamber (Neuroprobe, Cabin John, MD, USA) as previously described (Falk *et al.*, 1980). A 25 μl aliquot of either supernatant ($10^{-7}$ M *N*-formyl-methionyl-leucyl-phenylalanine) (FMLP) (Sigma), hu-rIL-8 (1–100 ng $ml^{-1}$), hu-rMCP-1 (1–100 ng $ml^{-1}$) or phosphate-buffered saline (PBS) was placed in triplicate lower wells of Boyden chambers. A 8 μm (for monocytes) or 3 μm (for neutrophils) pore size polycarbonate filter (Nucleopore, Pleasanton, CA, USA) was placed in the assembly and 50 μl ($1.5 \times 10^6$ cells $ml^{-1}$) of PBMCs, or PMNs was placed in each upper well. Chemotaxis chamber assemblies were incubated at 37°C in humidified 95% air/5% carbon dioxide for 1 h and filters were removed, fixed in 70% methanol and stained with Diff-Quik 1 and 2 (Kokusai Shiyaku, Kobe, Japan). Monocytes or PMNs that had migrated through onto the lower surface of the filter were counted under the microscope.

*Animal studies*

Seven-week-old male BALB/c *nu/nu* mice were purchased from Nihon Crea (Atsugi, Japan). Transplantation assays for each chemokine transfectant were performed successively in combination with CHO/neo as a control. Tumour cell injections were carried out using freshly prepared suspensions at a concentration of $1.5 \times 10^7$ cells $ml^{-1}$. The total number of

**Table I** Chemokine secretion after gene transfer into tumour

| *Tumour* | *Production*[a] *(ng ml*$^{-1}$ $10^{-6}$ *cells 24 h*$^{-1}$*, mean ± s.d)* | *Activity*[b] *(chemotactic index, mean ± s.d.)* |
|---|---|---|
| CHO/hu-IL-8 | 4 ± 1 | 2.5 ± 0.3* |
| CHO/hu-MIP-1α | 7 ± 1 | 3.4 ± 0.5** |
| CHO/hu-MCP-1 | 82 ± 18 | 4.7 ± 0.5** |
| CHO/mu-MIP-1α | NT | 5.1 ± 0.6** |
| CHO/mu-MIP-2 | NT | 5.8 ± 1.1** |
| CHO/mu-MCP-1 | NT | 4.6 ± 1.1** |
| CHO/neo | – | 1.1 ± 0.3 |
| medium | – | 1.0 |

[a]The amount of hu-IL-8 and hu-MCP-1 in culture supernatants was measured by radioimmunoassay, and hu-MIP-1α was assayed using an ELISA system as described in Materials and methods. [b]The chemotactic activity was measured using a multimicro-well Boyden chamber as described in Materials and methods. NT, not tested. *$P<0.01$ *vs* medium (Student's *t* -test). **$P<0.001$ *vs* medium (Student's *t*-test).

**Figure 2** Comparison of the growth of control and chemokine-transfected CHO clones. Groups of six to nine mice were given injections of $3 \times 10^6$ tumour cells. Tumour growth was monitored as described in Materials and methods. ●, chemokine transfectant; ○, control transfectant (CHO/neo). *$P<0.01$ *vs* CHO/neo. **$P<0.001$ *vs* CHO/neo.

tumour cells injected per animal was $3 \times 10^6$, except for mixed tumour transplantation assay (see below). All injections were performed s.c. in the right lower abdominal quadrant via 27 gauge needles. Tumour volumes were measured in $mm^3$ with a vernier caliper and recorded by the formula $(a \times b^2/2)$, where $a$ is the larger and $b$ is the smaller of the two dimensions. All animal experiments were conducted in accordance with Animal Care and Use Committee guidelines of Kureha Chemical Industry.

*Mixed tumour transplantation assay*

The mixed tumour transplantation assay was performed by mixing CHO/neo ($3 \times 10^6$ cells) with CHO/hu-IL-8 ($1 \times 10^6$ cells), or CHO/mu-MIP-1α ($1 \times 10^6$ cells) and injecting them into nude mice as described above.

*Histology*

Tissues at the site of tumour injection were embedded in OCT compound (Miles. Elkhart, IN, USA) and snap frozen in liquid nitrogen. Six micron cryostat sections were fixed in 90% ethyl alcohol and stained with haematoxylin and eosin.

*Statistical analysis*

Values in the table are expressed as the mean ± s.e. The significance of the differences was calculated using the Student's $t$-test. Values of $P<0.05$ were considered to be significant.

### Results

*Production of chemokine in transfectants*

Twelve independent G-418-resistant colonies from each transfection of chemokine expression plasmid were isolated and expanded. One clone showing the highest level of mRNA expression for chemokine was selected for further study by the RNA blotting method, as shown in Figure 1. The CHO cells transfected with vector alone (CHO/neo: control) did not express any cross-hybridising endogenous chemokine genes. Furthermore, the production of chemokine was confirmed by assays of chemotactic activity, ELISA and/or RIA (Table I). Individual transfectants secreted substantial levels of chemokine activity, while control transfectant produced no chemotactic factor.

*Inhibition of tumour growth in vivo*

The transfection and expression of chemokine by CHO cells did not alter their growth properties *in vitro* as assayed by doubling time or morphology (data not shown). Chemokine gene-transfected clones as well as parental CHO cells transfected with vector alone were tested for tumorigenicity by s.c. transplantation into the flank of immunoincompetent nude mice (T-cell deficient). Figure 2 shows representative results from three experiments of the *in vivo* growth rate of chemokine transfectants. CHO/hu-MIP-2, CHO/hu-MCP-1, and CHO/mu-MCP-1 showed almost the same growth rate as control cells (CHO/neo) *in vivo*, whereas hu-IL-8-, mu- and hu-MIP-1α-expressing clone (CHO/hu-IL-8, CHO/mu-MIP-1α and CHO/hu-MIP-1α) grew significantly more slowly than control cells (CHO/neo). Table II shows tumour incidence and final tumour weight as assayed at 22 days after transplantation. The production of hu-IL-8 or hu- or mu-MIP-1α by tumour cells was associated with markedly suppressed tumour growth in nude mice. The growth inhibition was 86.7% for CHO/hu-IL-8, 30.7% for CHO/hu-MIP-1α and 94.4% for CHO/mu-MIP-1α. Furthermore, tumour formation was totally prevented in four of seven mice in the case of CHO/hu-IL-8 and in four of nine mice for CHO/mu-MIP-1α. In contrast, secretion of hu-, mu-MCP-1 or mu-MIP-2 by tumour cells did not cause a reduction or prevention of tumour growth.

*Mixed tumour transplantation assay*

Additional insight into the mechanism by which secretion of hu-IL-8 or mu-MIP-1α by a tumour suppressed cell growth *in vivo* came from assays of injections of tumour mixtures in which 3:1 mixtures of CHO/neo and CHO/hu-IL-8, or CHO/mu-MIP-1α cells were injected into nude mice. Although CHO/neo-tumour arose in 100% of animals, tumour developed in seven of nine or six of nine animals injected with a mixture of CHO/neo and CHO/hu-IL-8 or of CHO/neo and CHO/mu-MIP-1α respectively, as shown in Table III.

*Histology at the site of tumour cell injection*

To elucidate the host cellular responses activated by chemokine production, histological analysis of the injection site was performed at 48 h after tumour cell ($3 \times 10^6$), challenge as shown in Figure 3. Few inflammatory cells infiltrated the CHO/neo injection site. In contrast, the inoculation site of CHO/hu-IL-8, CHO/hu-MIP-1α and CHO/mu-MIP-1α contained a marked cellular infiltrate composed predominantly of neutrophils, as well as necrotic destruction of tumour cells. In contrast, the cellular infiltrate of sites injected with CHO/hu-MCP-1, CHO/mu-MCP-1 and CHO/mu-MIP-2 was very similar to that of the control CHO/neo or CHO injected recipients.

### Discussion

Numerous papers have been published on the anti-tumour effects of transfection of tumour cells with cytokine genes (Colombo and Forni, 1994). Transfection with virtually almost all cytokine genes results in inhibition of tumour

**Table II** Tumorigenicity after chemokine gene transfer[a]

| *Tumour* | *Tumour weight (g)*[b] *(mean ± s.d.)* | *Tumour incidence*[b] |
|---|---|---|
| CHO/hu-IL-8 | 0.08 ± 0.04* (86.7)[c] | 4 7 |
| CHO/neo | 0.60 ± 0.35 | 7 7 |
| CHO/hu-MIP-1α | 0.70 ± 0.29** (30.7)[c] | 6 6 |
| CHO/neo | 1.01 ± 0.25 | 7 7 |
| CHO/hu-MCP-1 | 1.01 ± 0.06 | 7 7 |
| CHO/neo | 1.08 ± 0.11 | 7 7 |
| CHO/mu-MIP-1α | 0.05 ± 0.01* (94.4)[c] | 4 9 |
| CHO/neo | 1.07 ± 0.11 | 9 9 |
| CHO/mu-MIP-2 | 0.87 ± 0.35 | 8 8 |
| CHO/neo | 0.50 ± 0.30 | 8 8 |
| CHO/mu-MCP-1 | 0.50 ± 0.16 | 8 8 |
| CHO/neo | 0.72 ± 0.28 | 8 8 |

[a]Mice were injected s.c. with $3 \times 10^6$ cells of tumour. [b]Tumour weight and incidence (number of mice with tumour/number of mice injected) refer to day 22 after transplantation. [c]Growth inhibition (%). *$P<0.001$ *vs* CHO/neo (control) (Student's $t$-test). **$P<0.01$ *vs* CHO/neo (Student's $t$-test).

**Table III** The effect of chemokines in mixed tumour transplantation[a]

| *Tumour* | *Tumour incidence*[b] |
|---|---|
| CHO/neo alone | 9 9 |
| CHO/neo plus CHO/hu-IL-8 | 7 9 |
| CHO/neo plus CHO/mu-MIP-1α | 6 9 |

[a]Mice were injected s.c. with CHO/neo ($3 \times 10^6$ cells) alone, or a mixture of CHO/neo ($3 \times 10^6$ cells) and chemokine-producing CHO ($1 \times 10^6$ cells). [b]Tumour incidence (number of mice with tumour/number of mice injected) refer to day 22 after transplantation.

growth, mediated through infiltration of T lymphocytes and/or macrophages into the tumour site.

Ours is the first report demonstrating the inhibition of tumour growth in nude mice by secretion of IL-8 or MIP-1α, and this was accompanied by neutrophilic infiltration. Both purified IL-8 and MIP-1α have been reported to act on several types of immune cells (Oppenheim *et al*., 1991) but did not show tumour cell killing activity *in vitro*. On the basis of their *in vitro* properties (Wolpe and Cerami, 1989; Oppenheim *et al*., 1991), IL-8 and MIP-1α are reported to

**Figure 3** Cellular infiltrate at the injection site of chemokine-producing CHO cells. Nude mice were injected s.c. with 3 × $10^6$ cells. The mice were sacrificed 48 h later and the injection sites were processed for histological examination. All sections were stained with haematoxylin and eosin. Magnification × 200.

attract neutrophils and, perhaps, to activate anti-tumour properties of neutrophils. If neutrophils are responsible for tumour-growth suppression in nude mice, there are several possible mechanisms whereby IL-8- or MIP-1α-activated neutrophils might exert their effects. These chemokines activate neutrophils to release cytotoxic oxygen radicals and/or proteases, which could kill tumour cells. It is also possible for neutrophils to produce a soluble mediator of tumour cell killing such as TNF, IL-1 and IFNs in response to these chemokines, since neutrophils have been reported to be potent producers of cytokines (Lloyd and Oppenheim, 1992). Interestingly, mu-MIP-2, which has also been reported to be a chemokine for neutrophils *in vitro* (Anisowicz *et al.*, 1987), showed only a very low level of neutrophilic infiltration, similar to that of the controls, and had no anti-tumour activity. This was perhaps due to insufficient attraction of neutrophils by the MIP-2-transfected cells. Although two research groups have independently demonstrated MCP-1 (MCAF, JE) secretion by transfected tumour induced monocyte-mediated tumoricidal activity in syngeneic (Bottazi *et al.*, 1992) and nude mice (Rollins and Sunday, 1991), we did not observe the suppression of tumour growth and monocytic infiltration at the injection site of MCP-1-producing tumour cells. This discrepancy might derive from differences in the level of MCP-1 production by tumour cells. We may not have achieved optimal concentration of this chemokine for chemotactic effects on monocytes *in vivo*. It is also possible that CHO cells spontaneously produce certain cross species-reactive cytokines which may have influenced the chemotactic activities of chemokine produced by the transfectants.

We have observed that mu-MIP-1α had much more potent tumoricidal activity than hu-MIP-1α in nude mice, perhaps because of species differences. Histological examination demonstrated that mu-MIP-1α resulted in greater accumulation in mu-neutrophils than hu-MIP-1α. Nevertheless, hu-MIP-1α is quite cross species-reactive, as previously reported (Dunlop *et al.*, 1992). The mixed tumour transplantation assay demonstrated that the effects of hu-IL-8 and mu-MIP-1α were transmitted from producer to non-producer cells.

It was recently reported that gene transfer of IP-10, which is a member of the C-X-C chemokines superfamily, elicited a more potent host-mediated anti-tumour effect in syngeneic, immunocompetent mice than in immunoincompetent nude mice (Luster and Leder, 1993). The anti-tumour response of IP-10 is T lymphocyte dependent, not limited to secreting cells, and appears to be mediated by the recruitment of cell infiltrate composed of T lymphocytes *in vivo*. Since IL-8 is, also, a potent chemoattractant for T lymphocytes (Larsen *et al.*, 1989), an enhanced anti-tumour effect might be expected in syngeneic, immunocompetent mice, but this remains to be shown. On the other hand, MIP-1α is unlikely to show more potent anti-tumour effects in syngeneic than in nude mice, because the injection site (s.c.) of Lewis lung carcinoma cells transfected with mu-MIPα to syngeneic mice contained very few infiltrating T lymphocytes (data not shown).

Although IL-8 was originally identified as a neutrophil chemoattractant, subsequent work has revealed its multifunctionality, as is the case with most cytokines. IL-8 can induce the migration of some tumour cells (Wang *et al.*, 1990) and stimulate the growth of melanoma cells (Schadendorf *et al.*, 1993). IL-8 has also been shown to be an angiogenic factor released by activated macrophage (Koch *et al.*, 1992; Strieter *et al.*, 1992). Since cell migration, proliferation and angiogenesis are all essential components of the metastatic process (Fidler *et al.*, 1990), IL-8 expression by tumour cells may influence their metastatic capabilities. In fact, a recent paper has clearly demonstrated that the expression level of IL-8 correlated with the metastatic potential of human melanoma cells implanted into nude mice (Singh *et al.*, 1994). However, we have not observed metastatic behaviour in CHO cells transfected with IL-8 in nude mice (data not shown). These contradictory observations may be explained by differences in the metastatic potential of different tumour cell types. Factors in addition to IL-8 production might be required for metastasis of CHO tumour cells.

The fact that MIP-1α, a C-C chemokine, results in the accumulation of neutrophils, but not of monocytes, in our *in vivo* study was quite unexpected, because C-C chemokines have been thought to be predominantly chemotactic for monocytes (Oppenheim *et al.*, 1991).

The local injection of immunomodulating agents such as LPS and PSK (protein-bound polysaccharide) (Nakazato *et al.*, 1994), which are potent inducers of chemokines, at the tumour site (Matsushima *et al.*, 1988; Hirose *et al.*, 1990), could lead to tumour cell killing based on local chemokine secretion.

Chemokines may be useful clinically in combination with anti-cancer agents and/or other types of cytokines such as IL-2, IFNs, and CSFs, since they seem to have different anti-tumour mechanisms and to be well tolerated at high doses. This leads us to hypothesise that engineering tumour-infiltrating lymphocytes (TILs) to express a chemokine might provide synergistic local tumour cell killing.

**Acknowledgement**
We would like to express our sincere appreciation to Dr Joost J Oppenheim, Laboratory of Molecular Immunoregulation, Biological Response Modifiers Program, National Cancer Institute, USA for helpful discussion and criticism of this manuscript.

**References**

ANISOWICZ A, BARDWELL L AND SAGER R. (1987). Constitutive overexpression of a growth-regulated gene in transformed Chinese hamster and human cells. *Proc. Natl Acad. Sci. USA*, **84**, 7188–7192.

BLANKENSTEIN T, QIN Z, UBERLA K, MULLER W, ROSEN H, VOLK H-D AND DIAMANTSTEIN T. (1991). Tumor suppression after tumor cell-targeted tumor necrosis factor α gene transfer. *J. Exp. Med.*, **173**, 1047–1052.

BOTTAZI B, WALTER S, GOVONI D, COLOTTA F AND MANTOVANI A. (1992). Monocyte chemotactic cytokine gene transfer modulates macrophage infiltration, growth and susceptibility to IL-2 therapy of a murine melanoma. *J. Immunol.*, **148**, 1280–1285.

COLOMBO MP AND FORNI G. (1994). Cytokine gene transfer in tumor inhibition and tumor therapy: where are we now? *Immunol. Today*, **15**, 48–51.

COLOMBO MP, FERRARI G, STOPPACCIARO A, PARENZA M, RODOLFO M, MAVILIO F AND PARMIANI G. (1991). Granulocyte colony stimulating factor gene transfer suppresses tumorigenicity of a murine adenocarcinoma *in vivo*. *J. Exp. Med.*, **173**, 889–897.

COLUMBEC PT, AZHARI R, ELIZABETH M, LEVITSKY HI, LAZENBY A, LEONG K AND PARDOLL DM. (1993). Controlled release, biodegradable cytokine depots: a new approach in cancer vaccine design. *Cancer Res.*, **53**, 5841–5844.

DUNLOP DJ, WRIGHT EG, LORIMORE S, GRAHAM GJ, HOLYOAKA T, KERR, DJ, WOLPE SD AND PRAGNELL IB. (1992). Demonstration of stem cell inhibition and myeloprotective effects of SCI rhMIPIα *in vivo*. *Blood*, **79**, 2221–2225.

ENDO H, AKAHOSHI T, TAKAGISHI K, KASHIWAZAKI S AND MATSUSHIMA K. (1991). Elevation of interleukin-8 (IL-8) levels in joint fluids of patients with rheumatoid arthritis and induction by IL-8 of leukocyte infiltration and synovitis in rabbit joints. *Lymphokine Cytokine Res.*, **10**, 245–252.

FALK W, GOODWIN RH AND LEONARD EJ. (1980). A 48-well microchemotaxis assembly for rapid and accurate measurement of leukocyte migration. *J. Immunol. Methods*, **33**, 239–248.

FERRANTINI M. PROIETTI E. SANTODONATO L. GABRIELE L. PERETTI M. PLAVEC I. MEYER F. KAIDO T. GRESSER I AND BELARDELLI F. (1993). α1-Interferon gene transfer into metastatic friend leukemia cells abrogated tumorigenicity in immunocompetent mice: antitumor therapy by means of interferon-producing cells. *Cancer Res.*, **53**, 1107-1112.

FIDLER IJ. (1990). Critical factors in the biology of human cancer metastasis: twenty-eighth G.H.A. Clowes Memorial Award Lecture. *Cancer Res.*, **50**, 6130-6138.

FURUTANI Y. NOMURA H. NOTAKE M. OYAMADA Y. FUKUI T. YAMADA M. LARSEN CG. OPPENHEIM JJ AND MATSUSHIMA K. (1989). Cloning and sequencing of the cDNA for human monocyte chemotactic and activating factor (MCAF). *Biochem. Biophys. Res. Commun.*, **159**, 249-255.

GANSBACHER B. ZIER K. DANIELS B. CRONIN K. BANNERJI R AND GILBOA E. (1990). Interleukin 2 gene transfer into tumor cells abrogates tumorigenicity and induces protective immunity. *J. Exp. Med.*, **172**, 1217-1224.

GUNNING P. LEAVITT J. MUSCAT G AND KEDES L. (1987). A human β-actin expression vector system direct high level accumulation of antisense transcript. *Proc. Natl Acad. Sci. USA*, **84**, 4831-4835.

HIROSE K. ZACHARIAE COC. OPPENHEIM JJ AND MATSUSHIMA K. (1990). Induction of gene expression and production of immunomodulating cytokines by PSK in human peripheral blood mononuclear cells. *Lymphokine Res.*, **9**, 475-483.

HOCK H. DORSCH M. DIAMANTSTEIN T AND BLANKENSTEIN T. (1991). Interleukin 7 induces $CD4^+$ T cell-dependent tumor rejection. *J. Exp. Med.*, **174**, 1291-1298.

KAWAHARA RS AND DEUEL TF. (1989). Platelet-derived growth factor-inducible genes related to platelet factor 4. *J. Biol. Chem.*, **264**, 679-682.

KOCH AE. POLVERINI PJ. KUNKEL SL. HARLOW LA. DIPIETRO LA AND ELENA VM. (1992). Interleukin-8 as a macrophage derived mediator of angiogenesis. *Science*, **258**, 1798-1801.

LARSEN CG. ANDERSON AO. APPELLA E. OPPENHEIM JJ AND MATSUSHIMA K. (1989). Neutrophil activating protein (NAP-1) is also chemotactic for T lymphocytes. *Science*, **243**, 1464-1466.

LIPSON KE AND BASERGA R. (1989). Transcriptional activity of human thymidine kinase gene determined by a method using the polymerase chain reaction and an intron-specific probe. *Proc. Natl Acad. Sci. USA*, **86**, 9774-9784.

LLOYD AR AND OPPENHEIM JJ. (1992). Poly's lament: the neglected role of the polymorphonuclear neutrophil in the afferent limb of the immune response. *Immunol. Today*, **13**, 169-172.

LUSTER AD AND LEDER P. (1993). IP-10, a C-X-C chemokine, elicits a potent thymus-dependent antitumor response *in vivo*. *J. Exp. Med.*, **178**, 1057-1065.

LUSTER AD. UNKELESS JC AND RAVETCH JV. (1985). Gamma interferon transcriptionally regulates an early-response gene containing homology to platelet proteins. *Nature*, **315**, 672-676.

MATSUSHIMA K. AKAHOSHI T. YAMADA M. FURUTANI Y AND OPPENHEIM JJ. (1986). Properties of a specific interleukin 1 (IL-1) receptor on human Epstein-Barr virus transformed B lymphocytes: Identity of the receptor for IL-1α and IL-1β. *J. Immunol.*, **136**, 4496-4506.

MATSUSHIMA K. MORISHITA K. YOSHIMURA T. LAVU S. KOBAYASHI Y. LEE W. APPELLA E. KUNG HF. LEONARD EJ AND OPPENHEIM JJ. (1988). Molecular cloning of a human monocyte-derived neutrophil chemotactic factor (MDNCF) and the induction of MDNCF mRNA by interleukin 1 and tumor necrosis factor. *J. Exp. Med.*, **167**, 1883-1893.

MATSUSHIMA K. LARSEN CG. DUBOIS CG AND OPPENHEIM JJ. (1989). Purification and characterization of a novel monocyte chemotactic protein and activating factor produced by a human myelomonocytic cell line. *J. Exp. Med.*, **169**, 1485-1493.

MULLEN CA. COALE MM. LEVY AT. STETLER-STEVENSON WG. LIOTTA LA. BRANDT S AND BLAESE RM. (1992). Fibrosarcoma cells transduced with the IL-6 gene exhibit reduced tumorigenicity, increased immunogenicity, and decreased metastatic potential. *Cancer Res.*, **52**, 6020-6024.

NAKAZATO H. KOIKE A. SAJI S. OGAWA N AND SAKAMOTO J. (1994). Efficacy of immunochemotherapy as adjuvant treatment after curative resection of gastric cancer. *Lancet*, **343**, 1122-1126.

OBUKU K. FUKUDA M. MAEDA S AND SHIMADA K. (1986). A cDNA clone used to study mRNA inducible in human tonsillar lymphocytes by a tumor promoter. *J. Biochem.*, **99**, 885-894.

OPPENHEIM JJ. ZACHARIAE COC. MUKAIDA N AND MATSUSHIMA K. (1991). Properties of the novel proinflammatory supergene 'intercrine' cytokine family. *Annu. Rev. Immunol.*, **9**, 617-648.

ROLLINS BJ AND SUNDAY ME. (1991). Suppression of tumor formation *in vivo* by expression of the JE gene in malignant cells. *Mol. Cell. Biol.*, **11**, 3125-3131.

SCHADENDORF D. MOLLER A. ALGERMISSEN B. WORM M. STICHERLING M AND CZARNETZKI BM. (1993). IL-8 produced by human malignant melanoma cells *in vitro* is an essential autocrine growth factor. *J. Immunol.*, **151**, 2667-2675.

SCHALL TJ. BACON K. TOY KJ AND GOEDDEL DV. (1990). Selective attraction of monocytes and T lymphocytes of the memory phenotype by cytokine RANTES. *Nature*, **347**, 669-673.

SINGH RK. GUTMAN M. RADINSKY R. BUCANA CD AND FIDLER IJ. (1994). Expression of interleukin 8 correlates with the metastatic potential of human melanoma cells in nude mice. *Cancer Res.*, **54**, 3242-3247.

STRIETER RM. KUNKEL SL. ELNER VM. MARTONYI CL. KOCH AE. POLVERINI PJ AND ELNER SG. (1992). Interleukin-8: a corneal factor that induces neovascularization. *Am. J. Pathol.*, **141**, 1279-1284.

TEPPER RI. PATTENGALE PK AND LEDER P MURINE. (1989). Interleukin-4 displays potent anti-tumor activity *in vivo*. *Cell*, **57**, 503-512.

WANG JM. TARABOLETTI G.. MATSUSHIMA K. DAMME JV AND MANTOVANI A. (1990). Induction of hepatotactic migration of melanoma cells by neutrophil activating protein IL-8. *Biochem. Biophys. Res. Commun.*, **169**, 165-170.

WATANABE Y. KURIBAYASHI K. MIYATAKE S. NISHIHARA K. NAKAYAMA E. TANIYAMA T AND SAKATA T. (1989). Exogenous expression of mouse interferon gamma cDNA in mouse neuroblastoma C1300 cells results in reduced tumorigenicity by augmented anti-tumor immunity. *Proc. Natl Acad. Sci. USA*, **86**, 9456-9460.

WOLPE SD AND CERAMI A. (1989). Macrophage inflammatory proteins 1 and 2: members of a novel superfamily of cytokines. *FASEB J.*, **3**, 2565-2573.

WOLPE SD. DAVATELIS G. SHERRY B. BEUTLER B. HESSE SD AND CERAMI A. (1988). Macrophages secrete a novel heparin binding protein with inflammatory and neutrophil chemokinetic properties. *J. Exp. Med.*, **167**, 570-581.

WOLPE SD. SHERRY B. JUERS D. DAVATELIS G. YURT RW AND CERAMI A. (1989). Identification and characterization of macrophage inflammatory protein 2. *Proc. Natl Acad Sci USA*, **86**, 612-616.